# Evaluation of Styloid Process Elongation in Madinah, Saudi Arabia: A Retrospective Radiographic Investigation

**DOI:** 10.7759/cureus.53136

**Published:** 2024-01-29

**Authors:** Muhannad Kaaki, Muath S Alassaf, Albraa Alolayan, Esam S Almuzaini, Ahmed K Alsaeedi, Abdulsamad Habeeb, Shadia Abdelhameed N Elsayed

**Affiliations:** 1 Oral Basic and Clinical Sciences, Taibah University, Madinah, SAU; 2 Orthodontics and Dentofacial Orthopedics, Taibah University, Madinah, SAU; 3 Oral and Maxillofacial Surgery, Taibah University, Madinah, SAU; 4 Dental Education, Taibah University, Madinah, SAU; 5 General Dentistry, Taibah University, Madinah, SAU; 6 Oral Surgery, Taibah University, Madinah, SAU

**Keywords:** elongated styloid syndrome, panoramic radiograph, neck pain, eagle syndrome, styloid process, styloid

## Abstract

Objectives: This study aimed to identify the prevalence of an elongated styloid process and analyze the presence of its calcification in the Saudi population using panoramic radiographs.

Methods: The Taibah Outpatient Dental Clinic's OPG radiographs for 962 patients who attended screening clinics between December 2022 and October 2023 were all included in the study. Patients' demographics, such as age, gender, and nationality, as well as radiological data, were included in the following study variables: the presence of an elongated styloid on both sides of a panoramic radiograph, right side styloid length, left side styloid length, right side distal end thickness, and left side distal end thickness.

Results: The study evaluated 438 (45.5%) processes found in individuals aged 16-80 years old. The elongated process length ranged from 30.0 to 40.1 mm, and the diameter ranged from 0.81 to 7.79 mm at the origin to 0.56-3.79 mm at the end. There was no statistically significant difference in process length across genders or age groups. The diameters of the styloid bones on the left side vary significantly across genders at the start and completion of the process.

Conclusion: The prevalence of elongated styloids in the studied population was 4.26%. The radiological evaluation of the styloid process is a crucial stage in dental surgery planning.

## Introduction

A styloid process is a cartilaginous bone arising from the posterior part of the temporal bone [1,2]. Reichert's cartilage of the second pharyngeal arch developed the stylohyoid apparatus, which includes the styloid process, the stylohyoid ligament, and the stylohyoid process's horn of the hyoid bone [3]. Numerous critical neurovascular structures, including the glossopharyngeal, vagus, accessory, and hypoglossal nerves, run medially to the styloid process, the internal carotid artery, and the internal jugular vein. The styloid process has a cylindrical form and a typical length of 20-30 mm [4,5]. The elongation of a styloid process greater than 30 mm is clinically significant because it may compress adjacent vital structures, resulting in various clinical symptoms, including dysphagia, otalgia, orofacial pain, and a feeling of a foreign object in the throat. These symptoms are all common to Eagle syndrome [6,7]. Panoramic radiography or computed tomography are the preferred methods for detecting any elongation or calcification related to the styloid process since they may show both sides and help compare the lengths of the right and left side styloid processes in the same scan [2,3]. Computed tomography is currently considered the primary source for investigations on calibration [2,3,8,9]. In contrast, panoramic radiographs are considered a routine diagnostic aid used in many dental facilities for diagnosis [10]. It also displays styloid processes with much lower radiation dose and at lower cost [11]. Identifying the prevalence of patients suffering from the elongated styloid processes in the Madinah community, Saudi Arabia, had significant clinical implications for oral and maxillofacial surgeons. For this reason, this study used panoramic radiographs to assess the prevalence of an extended styloid process in the Saudi population to provide answers to the following question: how common is the styloid process elongation in Madinah, Saudi Arabia?

## Materials and methods

Study design

A descriptive cross-sectional radiographic study was conducted to assess the length and status of a styloid process using panoramic radiographs. The study proposal was approved by the Taibah University Dental College's Ethical Committee (TUCD/REC140623/MMKAAKI). All patients were kept anonymous, and radiographs were taken from the R4 system (PracticeWorks Ltd., Atlanta, GA). The study was a retrospective radiographic study with no risks or problems.

Study setting

Ortho-pan-tomogram (OPG) radiographs were collected from the archived patient's records to investigate and assess the apparent length of the styloid process using Kodak R4 software provided with the respective machines at the outpatient Taibah dental clinics.

The Taibah Outpatient Dental Clinic's convenient OPG radiographs for patients who attended screening clinics between December 2022 and October 2023 were all included in the study. The study included radiographs that show styloid processes for patients in the age group above 18 years old. Exclusion criteria included radiographs with blurred images or magnification errors, history of fracture styloid, and pathology that impeded the calibration process.

Sample size

The nonrandom convenient sample of records between December 2022 and October 2023 was used. A total of 962 OPGs available radiographs were screened to highlight and detect any abnormality seen regarding the styloid process.

Variables

**Figure 1:**
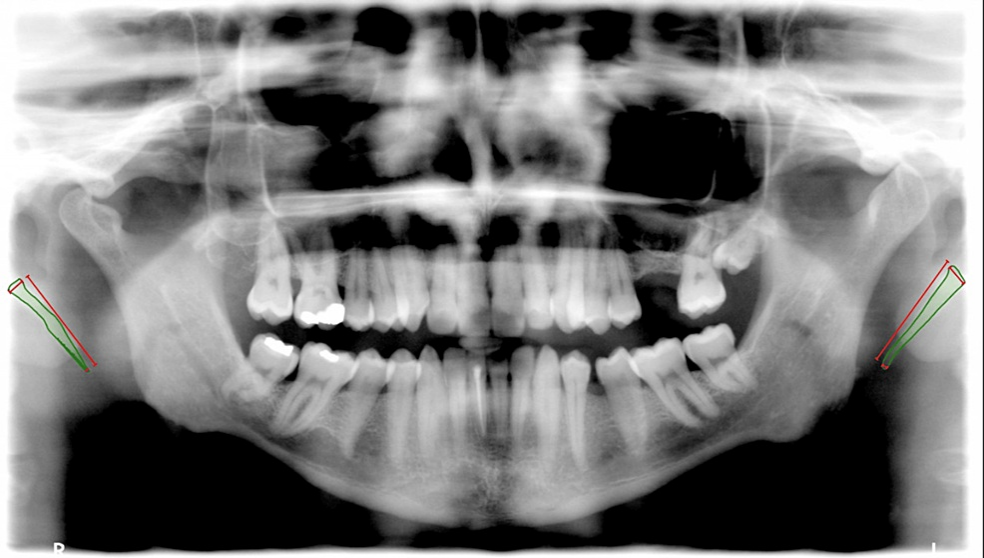
Measurement methods for the styloid process length and diameter at the origin and the end on the panoramic radiograph Green indicates the outline of the styloid process, and red indicates the length and the diameter at the origin and end of the styloid process.

The study variables included the patient's demographics, which included age, gender, and nationality, and radiographic parameters included the presence of the styloid on the panoramic radiograph on both sides, length of the styloid on the right side, length of the styloid on the left side, thickness at the distal end on the right side, and thickness at the distal end on the left side. The measurement method is shown in Figure 1.

Calibration method

Two examiners were responsible for the measurement. A training session was applied to both examiners prior to the beginning by an expert radiologist to unite the calibration on panoramic radiographs. All images were measured with the same setting area on the dark arena with a mutual display setting on the monitor.

Data analysis

Statistical Product and Service Solutions (SPSS, version 16; IBM SPSS Statistics for Windows, Armonk) was used for statistical analysis. Sample characteristics were reported using descriptive analysis. Continuous variables were presented as mean with standard deviations (M+SD). Analysis of the qualitative variables was done as frequency and percentages. ‎‏In the comparison process between groups, appropriate parametric (e.g., Student t-test) and non-parametric (e.g., chi-squared test (x2)) tests were used. The significance level was set at p-value ≤ 0.05.

## Results

The present study included 962 OPG radiographs that were evaluated for the presence of the styloid process. The study included 438 panoramic radiographs with styloid processes. These were found in patients aged between 16 and 80 years old, with a mean of 42.76 (±14.78).

The styloid process was found on panoramic radiographs in 438 (45.5%) of the investigated cases. Males had the process 333 (76%) more significantly than females (p=0.019) (Table 1). Age-wise, styloid processes appeared more in panoramic radiographs of patients in the age range of 20-50 years. The styloid process appeared on both sides in most cases (307), while, on one side, on the right or left, in 131 cases. Noteworthy is that, in 54.5% of the cases, the styloid process was not shown in the panoramic radiograph. There was no statistically significant difference in process length between genders or age groups (Table 2).

**Table 1:** Presence of the styloid process on the panoramic radiograph according to gender and age presented as frequency and percentage (%) (n=962) * Significant at level 0.05 or less

Variable		Both sides	Right side	Left side	Not present	Total	P-value
Gender	Female	229 (23.8%)	58 (6.0%)	46 (4.8%)	437 (45.4%)	770 (80.0%)	0.019*
	Male	78 (8.1%)	17 (1.8%)	10 (1.0%)	87 (9.0%)	192 (19.9%)	
Age Group (Years)	<20	16 (1.7%)	7 (0.7%)	2 (0.2%)	132 (13.7%)	157 (16.3%)	0.001*
	20-29	82 (8.5%)	23 (2.4%)	18 (1.9%)	170 (17.7%)	293 (30.4%)	
	30-39	61 (6.3%)	21 (2.2%)	10 (1.0%)	96 (10.0%)	188 (19.5%)	
	40-49	69 (7.2%)	10 (1.0%)	17 (1.8%)	62 (6.4%)	158 (16.4%)	
	50-59	47 (4.9%)	6 (0.6%)	5 (0.5%)	40 (4.2%)	98 (10.2%)	
	60-69	21 (2.2%)	4 (0.4%)	2 (0.2%)	16 (1.7%)	43 (4.5%)	
	>70	11 (1.1%)	4 (0.4%)	2 (0.2%)	8 (0.8%)	25 (2.6%)	
Total		307 (31.9%)	75 (7.8%)	56 (5.8%)	524 (54.5%)	962 (100.0%)	

**Table 2:** Mean and standard deviation (SD) measurements of the styloid process length in the studied population according to gender and age groups (n=745) All measurements are in millimeters and reported as mean and (standard deviation). * Significant at level 0.05 or less

Variable		Right side	Left side
Gender	Female	19.66 (7.1)	20.12 (6.9)
	Male	19.19 (6.7)	20.02 (7.3)
	P-value	0.621	0.773
Age Group (Years)	<20	17.02 (8.4)	18.44 (9.1)
	20-29	17.49 (6.2)	19.06 (7.0)
	30-39	20.22 (6.7)	20.21 (6.7)
	40-49	21.16 (7.4)	20.34 (6.7)
	50-59	19.85 (6.7)	21.41 (6.4)
	60-69	20.46 (6.9)	20.08 (6.3)
	>70	21.19 (7.5)	20.68 (9.1)
	P-value	0.026*	0.164

The styloid bone diameters differed significantly between the genders at the origin and end of the process (Table 3). However, no statistically significant differences were found among the age groups for the diameter at the origin or end of the styloid processes.

**Table 3:** Comparing the diameter at the origin and end of the styloid process in the studied population as mean with standard deviation (SD) (n=745) All measurements are in millimeters and reported as mean and (standard deviation). * Significant at level 0.05 or less

Variable		Right-side origin	Right-side end	Left-side origin	Left-side end
Gender	Female	2.3 (1.1)	1.6 (1.1)	2.4 (1.1)	1.1 (0.6)
	Male	2.7 (1.2)	1.5 (0.7)	2.7 (1.3)	1.7 (1.9)
	P-value	0.009*	0.001*	0.223	0.001*
Age Group (Years)	<20	2.3 (1.3)	3.6 (1.0)	2.2 (1.2)	1.2 (0.8)
	20-29	2.4 (1.3)	1.3 (0.7)	2.5 (1.4)	1.4 (1.6)
	30-39	2.5 (1.0)	1.3 (0.7)	2.4 (1.1)	1.3 (1.4)
	40-49	2.4 (0.9)	1.3 (0.6)	2.6 (1.2)	1.3 (0.7)
	50-59	2.8 (1.4)	1.2 (15.4)	2.6 (1.3)	1.2 (0.6)
	60-69	2.2 (1.1)	3.5 (0.6)	2.4 (0.9)	1.2 (0.8)
	>70	2.1 (1.0)	1.1 (0.5)	2.5 (1.0)	0.8 (0.5)
	P-value	0.430	0.273	0.549	0.606

Elongated styloid processes were found in 41 cases. The maximum length was 50.13 mm and 46.52 mm on the right and left sides, respectively. The characteristics of the elongated styloid processes are shown in Table 4.

**Table 4:** Characteristics of the elongated styloid cases from the included cases (n=41) All measurements are in millimeters and reported as mean and standard deviation.

	N	Minimum	Maximum	Mean	Std. Deviation
Age	41	16	80	42.76	14.78
Right Length	25	30	50.13	33.99	4.27
Right Diameter Origin	25	0.81	5.06	2.69	1.14
Right Diameter End	25	0.56	3.79	1.39	0.71
Left Length	24	30.04	46.52	34.73	4.74
Left Diameter Origin	24	1.42	7.79	3.04	1.40
Left Diameter End	24	0.65	3.61	1.37	0.71

## Discussion

The present study aimed to identify the prevalence of the elongated styloid process in the Madinah population using digitalized panoramic radiographs. The styloid process is a prominent bony protrusion that originates from the inferior section of the petrous temporal bone near the base of the skull [12,13]. Assiri et al. reported that the styloid process is present in 3.3%-84.4% of the Saudi population overall and that 4-10.3% of individuals with the elongated styloid process have eagle's syndrome [14].

The length of the styloid process can be influenced by developmental processes, hormone effects, and hereditary factors [9,15]. Furthermore, investigations have revealed that the length of the styloid process increases with age, suggesting that aging plays a role in this phenomenon; however, a study by Gokce et al. reported that age had no role in process elongation, and this goes in accordance with the present results [5].

The difference in the styloid process length between males and females can also be used for forensic identification, where the gender needs to be determined for research purposes [16,17]. Measuring the length of the styloid process can provide valuable information for narrowing down potential matches in missing persons cases or identifying unknown human remains [18]. According to the current study, there was a non-significant difference between genders. Further research is needed to fully understand the mechanisms and factors involved in the difference in styloid process length between genders for various medical and forensic applications [16]. Additionally, during clinical diagnosis and treatment, knowing the difference in styloid process length between males and females can aid in the differentiation of certain diseases [19] and help clinicians assess the likelihood of the syndrome in male and female patients [20].

The elongation of the styloid process causes symptoms in Eagle syndrome, such as throat pain and difficulty swallowing. Additionally, the elongated styloid process has implications in dental surgery [4]. During dental surgery, the elongated styloid process can pose challenges and potential risks to patients. The proximity of the elongated styloid process to nerves, muscles, and ligaments in the head and neck region increases the risk of complications during dental procedures such as tooth extractions, implant placements, or orthognathic surgery [21,22].

Careful assessment of the patient's radiographs and clinical examination should be done prior to dental surgery to identify any elongated styloid process. In order to determine the most appropriate surgical approach and technique to minimize the risk of injury or complications [20], radiological assessment of the styloid process is an essential step in dental surgery planning [23], and panoramic radiographs are reported to be the best substitute for expensive CT for assessment of the styloid process [1]. Surgical management of the symptomatic elongated process could include styloid resection through an extraoral approach or with simultaneous tonsillectomy [7].

In addition, neurological symptoms associated with an elongated styloid process can impact dental surgery. Patients with neurological symptoms such as migraines, headaches, or trigeminal neuralgia caused by irritation of the sympathetic nerve plexus may experience increased sensitivity or pain during dental procedures. Moreover, the elongated styloid process can lead to difficulties in local anesthesia administration due to altered anatomy and potential nerve impingement [19,20,24].

Limitations of the study

In this study, several limitations are noteworthy. The single-center approach limits the generalizability of the findings, as it may not accurately represent the wider population. Panoramic radiography, being a 2D imaging method, inherently struggles with accurately depicting three-dimensional structures, leading to potential distortions. The lack of comparative data with more advanced imaging techniques, such as 3D CBCT, limits the assessment of the panoramic radiograph's accuracy. Technique sensitivity, such as patient positioning and machine calibration, further impacts the reliability of the results.

## Conclusions

The present study is an important evaluation of the styloid process in the Madinah population, which is an important stage in the planning of dental surgery and the avoidance of elongated process complications. This study contributes to the body of knowledge necessary for improved clinical practice and provides a basis for further research into the developmental, pathological, and treatment aspects of the elongated styloid process. Understanding the prevalence and implications of this condition in the Madinah region will aid in better patient care, more accurate diagnostic procedures, and the development of targeted treatment plans.
